# Supervised and Weakly Supervised Deep Learning for Segmentation and Counting of Cotton Bolls Using Proximal Imagery

**DOI:** 10.3390/s22103688

**Published:** 2022-05-12

**Authors:** Shrinidhi Adke, Changying Li, Khaled M. Rasheed, Frederick W. Maier

**Affiliations:** 1 Institute of Artificial Intelligence, University of Georgia, Athens, GA 30602, USA; shrinidhi.adke@gmail.com (S.A.); khaled@uga.edu (K.M.R.); fmaier@uga.edu (F.W.M.); 2 Bio-Sensing and Instrumentation Laboratory, College of Engineering, University of Georgia, Athens, GA 30602, USA; 3 Phenomics and Plant Robotics Center, University of Georgia, Athens, GA 30602, USA

**Keywords:** cotton phenotyping, boll counting, supervised learning, mask R-CNN, weakly supervised learning

## Abstract

The total boll count from a plant is one of the most important phenotypic traits for cotton breeding and is also an important factor for growers to estimate the final yield. With the recent advances in deep learning, many supervised learning approaches have been implemented to perform phenotypic trait measurement from images for various crops, but few studies have been conducted to count cotton bolls from field images. Supervised learning models require a vast number of annotated images for training, which has become a bottleneck for machine learning model development. The goal of this study is to develop both fully supervised and weakly supervised deep learning models to segment and count cotton bolls from proximal imagery. A total of 290 RGB images of cotton plants from both potted (indoor and outdoor) and in-field settings were taken by consumer-grade cameras and the raw images were divided into 4350 image tiles for further model training and testing. Two supervised models (Mask R-CNN and S-Count) and two weakly supervised approaches (WS-Count and CountSeg) were compared in terms of boll count accuracy and annotation costs. The results revealed that the weakly supervised counting approaches performed well with RMSE values of 1.826 and 1.284 for WS-Count and CountSeg, respectively, whereas the fully supervised models achieve RMSE values of 1.181 and 1.175 for S-Count and Mask R-CNN, respectively, when the number of bolls in an image patch is less than 10. In terms of data annotation costs, the weakly supervised approaches were at least 10 times more cost efficient than the supervised approach for boll counting. In the future, the deep learning models developed in this study can be extended to other plant organs, such as main stalks, nodes, and primary and secondary branches. Both the supervised and weakly supervised deep learning models for boll counting with low-cost RGB images can be used by cotton breeders, physiologists, and growers alike to improve crop breeding and yield estimation.

## 1. Introduction

Cotton (*Gossypium hirstum* L.) is one of the most important cash crops in the world [1]. To improve the crop in terms of fiber yield and quality, cotton breeders need to measure plant phenotypic traits, and one of the most important traits is the total boll count. In addition to being valuable to breeding programs, boll count is also important to growers because it is the primary indicator of potential yield from the field. Furthermore, boll count can provide a good understanding about the growth conditions of the crop, which can lead growers to make crucial crop management decisions such as the timing of harvest [2]. It is an arduous task, however, to manually count cotton bolls accurately in the field and it even becomes impossible when a large number of plants (e.g., over a thousand plants with 40–50 bolls per plant) are involved. The cotton plant has a complex architecture, and bolls are distributed throughout the plant, making the counting task even more difficult. Traditionally, cotton breeders are only able to measure a limited number of samples to infer the larger population, which does not fully consider the variation within the population and impedes breeding programs.

To address this phenotyping bottleneck, researchers have been developing non-destructive high throughput phenotyping (HTP) techniques to automate the phenotyping tasks with less time and labor requirements [3,4]. In particular, computer vision used with deep learning has been playing an increasingly important role in HTP in several areas [5,6], such as plant disease classification [7,8], yield prediction [9], and plant organ detection and counting [10]. For example, Jiang et al. [11] developed an imaging system by leveraging deep learning object detection models to detect and count emerging cotton blooms that can characterize flowering patterns efficiently. A multi-object tracking approach was developed by the same group to count cotton seedlings and flowers from video frames over time [12,13,14]. There are a few studies on counting cotton bolls from proximal images. For instance, Sun et al. [15] implemented a boll recognition and counting pipeline based on traditional image-processing algorithms, achieving an accuracy of 84.6% in boll counting. Sun et al. [16,17] further demonstrated that machine learning models using hand-crafted features can segment cotton bolls from the 3D point cloud data with promising results. However, 3D point cloud data collection is more time consuming and costly than RGB images. Li et al. [18] leveraged the unsupervised clustering methods and region-based semantic segmentation with random forest to detect in-field cotton bolls with a best case segmentation accuracy of 97% for forward-facing images. However, this study only provided the number of superpixels for cotton bolls and did not provide the boll count from each plant. It would be desirable to leverage the deep learning approaches to count cotton boll numbers without hand-crafted features.

To make the deep learning model effective, the models have to be trained using a large number of annotated training data to learn diverse features in the data and reduce overfitting. However, annotation or labeling of training data in either bounding boxes or polygons is time consuming. In the case of cotton plants, a single plant in a 2D image typically contains 40 to 50 boll instance masks. These masks represent bolls with irregularly shaped polygons that require intricate annotations and consume at least 40 seconds for each mask and around 30 min per image for a skilled annotator. There are few public datasets available for the agricultural domain and most researchers have to label their training data from scratch. Furthermore, precise annotation of the training data in plant phenotyping has been a challenge because of factors such as domain knowledge, multi-modal input data, and application specific annotations. The burden of annotation on HTP researchers needs to be addressed to produce high quality deep learning models.

One promising way of addressing the data annotation burden is to provide weak supervision, either with partially annotated data or with pseudo-labels obtained from lower-level (image-level class annotations) labels. With the introduction of complex loss functions and processing, intermediate outputs can be used to perform image recognition tasks using the activation layer output and partially annotated datasets (only a subset of the objects in an image are labeled). These techniques have been used to perform advanced tasks, such as object instance counting and segmentation, which achieve comparable results to supervised methods [19]. When the lower-level labels are available, many approaches are available to localize and detect object instances [20] that leverage two prominent paradigms: multiple-instance learning (MIL) [21] and class feature activation maps (CAMs) [22]. MIL is based on learning object instances from positive (one or more instances present) or negative (no instances) bins of data samples, and this approach has been used widely for weakly supervised object detection [23,24]. CAMs use intermediate feature maps from the classifier’s activation layers along with pseudo-label generation, and the method has gained popularity due to its versatility for object detection as well as for instance segmentation [25,26,27].

Following the success of weakly supervised approaches for detecting and segmenting object instances, the approach is gaining researchers’ interest in medical and cellular biology fields, in which the annotated data are difficult to produce [28,29]. Recently, researchers have begun to explore weakly supervised models to reduce the annotation efforts in a variety of applications in the agricultural domain, including disease classification, yield estimation, and plant organ counting. For instance, Bollis et al. [30] designed a CNN-based algorithm to select automatically the regions of interest (ROI) from citrus fruit that were damaged by pests and diseases. The algorithm uses the MIL paradigm to classify those crops with the help of saliency maps, which significantly reduced the annotation costs. Ghosal et al. [31] performed head detection and counting to understand the relation between the phenotypic and genotypic traits of the sorghum crop. They demonstrated that it was possible to alleviate the annotation costs with the help of a partially annotated dataset in a weakly supervised learning setting without compromising the final model performance. Another weakly supervised counting network, PSSNet [32], used point-supervision to segment and count trees in aerial images and convert feature maps to masks. This network outperformed the state-of-the-art methods in most of the challenging conditions and greatly reduced human labor by generating masks automatically. As discussed previously, these approaches used either CAMs or MIL paradigms, but Yu et al. [33] proposed an approach combining both MIL and CAMs for Minirhizotron image segmentation, which outperformed standard weakly supervised semantic segmentation frameworks. Bellocchio et al. [34] proposed a novel fruit counting method for yield estimation based on spatial consistency loss, which falls under MIL because of the nature of the weak learning provided to the model. This weakly supervised counting (WS-Count) architecture performed exceptionally well in high density fruit counting and was able to achieve a performance similar to its fully supervised counterparts. Thus, for the boll counting application, we chose WS-Count as one of the frameworks to study in detail.

To the best of our knowledge, there are neither applications of supervised deep learning models nor weakly supervised learning methods to count the number of cotton bolls in a single plant using proximal RGB imagery. In particular, leveraging a weakly supervised paradigm with partial or low-level annotations from proximal imagery would help alleviate the annotation burden. Furthermore, there is a lack of understanding of the performance difference between supervised and weakly supervised approaches for cotton boll detection and counting. To address these gaps, the overall goal of this study is to segment and count cotton bolls from proximal imagery using supervised and weakly supervised deep learning with limited annotated data for training. The specific objectives of this study are to:

1. Train and test fully supervised deep learning models to segment cotton bolls from both indoor and infield images;
2. Develop weakly supervised methods based on class activation maps and multi-instance learning to segment cotton bolls from both indoor and infield images;
3. Compare the supervised and weakly supervised methods in terms of their performance on boll counting and annotation efficiency.

## 2. Materials and Methods

### 2.1. Data Source and Pre-Screening

The dataset used for this study consisted of RGB images of cotton plant from both potted (indoor and outdoor) and in-field settings, which reflected variations in plant conditions and background. The images were taken using hand-held consumer grade cameras. Pre-screening of data was performed to select good quality images, resulting in 290 images with resolutions ranging from 800 to 4000 pixels across both dimensions. During the initial experimentation with images at these resolutions, it was observed that if a single boll instance occupied only a very small area compared to an entire image, weakly supervised models would under-count the number of bolls. The object resolution in an image along with the pixel density for a single instance became an important factor in the learning process. It was also observed that at a certain point the model was not able to count more than a certain number of bolls, termed as the model’s subitizing range.

To make the boll number and resolution within each image conducive to the models, the entire dataset of 290 images was pre-processed to generate smaller, uniform image tiles containing fewer cotton bolls. Furthermore, 285 out of 290 images were selected to generate the training and validation sets with a uniform image size. The remaining 5 images with variable features were held out to test the entire processing pipeline. Table 1 summarizes this dataset and corresponding boll counts per image. The background and other adjacent cotton plants (if any) were cropped out to focus on the subject plant and reduce the noisy data while training. Furthermore, a fixed input image window of 500 × 500 was chosen to train the models and thus the images were cropped with zero padding along the borders. This resulted in 4266 image tiles from 285 images and 84 tiles from 5 held-out images, all with fixed size of 500 × 500 pixels.

### 2.2. Annotation Approaches

The cotton boll instances to be detected are complex in nature, with variable sizes, shapes, and appearances. In the dataset described above, an average cotton plant consisted of 35 bolls. To train the supervised models, all the ground truth object instances were annotated with pixel masks, also known as pixel-wise annotations or mask annotations. Each object instance in the image was annotated along its contour and the pixels enclosed in it represented the instance mask. The mask annotations demanded highly skilled annotators and their inexhaustible efforts both in terms of precision and time. The mask labels for bolls were obtained from 350 image tiles from Table 1 and annotated with additional instance masks. Furthermore, 300 images from the above training set were used for training and 50 from the testing set were used as a validation set.

**Figure 1:**
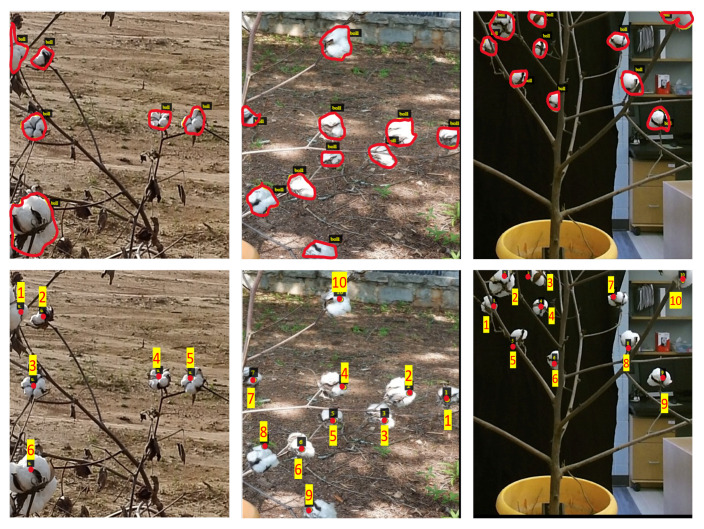
Annotation Types. Two main types of annotations were used in this study. The top row represents instance masks with class labels, whereas the bottom row represents point labels for the same image with the boll ID (the red numbers). The first column shows a sample tile from in-field plant image, whereas the second and third column show potted plants in outdoor and indoor conditions, respectively.

However, weakly supervised models require less annotation effort to achieve the same objective. Instead of providing total object count as a ground truth, weakly supervised methods for the counting task were trained using a classification or class-level annotations. This reduced the annotation efforts significantly, as the annotator was required to detect the presence of any single instance and give the classification label as class “present” or “absent”. Image tiles with “present” labels are further annotated using point labels. In point annotations [35], each boll instance was marked in the image with a point. Although only the boll presence label is required for learning, point labels help maintain the ground truth count for the image. Figure 1 shows the basic difference between the mask and point labels, representing one sample each from field data, potted outdoor data, and potted indoor data. In this study, all the annotations were performed using the VGG Image Annotator online tool [36].

Table 2 presents a summary of the total 4350 image tiles that were annotated with point annotations for six categories of boll counts. The images that do not contain bolls (background or other plant parts) were kept as negative category images, i.e., class label = 0. Even with pre-processing, in certain images the presence of background bolls from nearby plants contributed to noisy boll count. Following the literature for weakly supervised counting, a subitizing range was considered as [0, 10] and range [11, 15] was categorized as a challenging example. The image tiles containing over 15 bolls were discarded because most of the bolls were from the background.

Furthermore, as Table 2 shows, images with boll count in the range [1, 5] dominate the dataset (close to 50%) and this imbalance proved to be a limiting factor for the weakly supervised models. To avoid this imbalance, the 3712 training images were augmented manually with 1758 randomly sampled images from range [6, 15] by applying 90°, 180°, and 270° rotations. This training set with 5470 image tiles was used as the final dataset for weakly supervised counting.

### 2.3. Fully Supervised Learning Approaches

#### 2.3.1. Mask R-CNN

Mask R-CNN is considered one of the best performing instance segmentation models and was therefore chosen as one of the supervised models [37]. Mask R-CNN operates on the regions proposed by the region proposal network (RPN) to predict the class label, bounding box, and corresponding instance pixel masks from multiple regions of interest (ROIs). The choice of Mask R-CNN was inspired by the flexibility this method offers in terms of output. It can produce both instance masks and bounding boxes. In addition, it has been proven superior at localization tasks both in terms of precision and accuracy.

#### 2.3.2. Supervised Count Regression: S-Count

The S-Count network is an end-to-end fully supervised method that was trained as a regression model for counting bolls from the image tile. The network consists of ResNet-101 as the feature extractor without the final fully connected layer, which was replaced with an additional 1 × 1 convolution layer with *N* filters. This produces **N** filter maps which were treated as output response maps and fed to the fully connected layer to regress the boll count. The choice of *N* varies depending on the dataset. In this case, both *N* = 6 and *N* = 8 were experimented with variable random seed value to initialize the weights and data samples. Except the output layer, all layers have ReLU activation with batch normalization layers. Finally, the network is optimized with standard mean squared error (MSE) loss.

### 2.4. Weakly Supervised Learning

#### 2.4.1. MIL-CAM Based Weakly Supervised Counting: WS-Count

**Figure 2:**
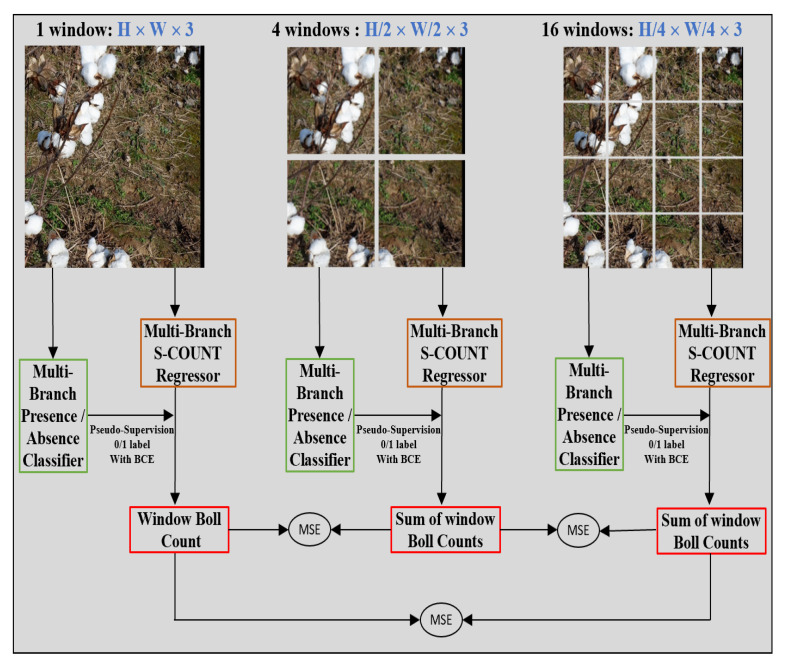
Schematic representation of WS-Count architecture. An image is divided into 4 windows and then further divided into 16 windows. A total of 21 images are passed to the main two networks that are responsible for boll counting. Presence Absence Classifier (PAC) detects the presence of boll in the patch and thus provides a weak supervision for the regression network, whereas the counting network (S-Count) estimates a boll count for that patch with the help of additional fully connected layers. The processing of 21 image patches in parallel makes the individual PAC and S-Count networks multi-branched (MB) and predicts unique output count for each of the 21 patches. The count predictions are kept in accordance with the classifier supervision and the total count loss is optimized through all the image levels.

Weakly Supervised Counting (WS-Count) [34] is a promising weakly supervised architecture for counting based on multi-instance learning (MIL) and class activation maps (CAMs). Its architecture is comprised of two sub networks: a presence-absence classifier and a regression network to produce the final count (Figure 2). The presence-absence classifier (binary classifier) takes the image as an input to detect the presence of a class in it and is trained in a supervised manner using the classification labels. The second part is the counting branch that regresses the object count based on the feature map and it is trained using the output of the first presence-absence classifier.

During the training of the WS-Count network, the input image was processed at three different scales (Figure 2): the original image tile scale, a quarter scale (the image tile is evenly divided into 4 sub-windows), and a 1/16 scale (the image tile is evenly divided into 16 sub-windows). This scaling makes this architecture a multi-branch (MB) network with a total of 21 branches being trained simultaneously. Each branch processes a sub-window and is weakly supervised by the PAC prediction of the same sub-window to produce the count. The learning objective for this method is to minimize the classifier consistency loss LPAC-C and a spatial consistency loss LSP-C. Classifier consistency loss maintains the coherence between classifier output and counts of each branch. Ideally, if the classifier predicts the presence of boll then the count network should produce a count greater than zero and vice versa. The main purpose of spatial consistency loss is to bring consistency between the total count at three different scales. A combined loss function was optimized during the training of the WS-count multi-branch network (Equation (1)).

(1) $$L_{\mathrm{WS}-\mathrm{Count}}=L_{\mathrm{PAC}-C}+L_{\mathrm{SP}-C}$$

#### 2.4.2. CAM Based Counting with Partial Labels: CountSeg

**Figure 3:**
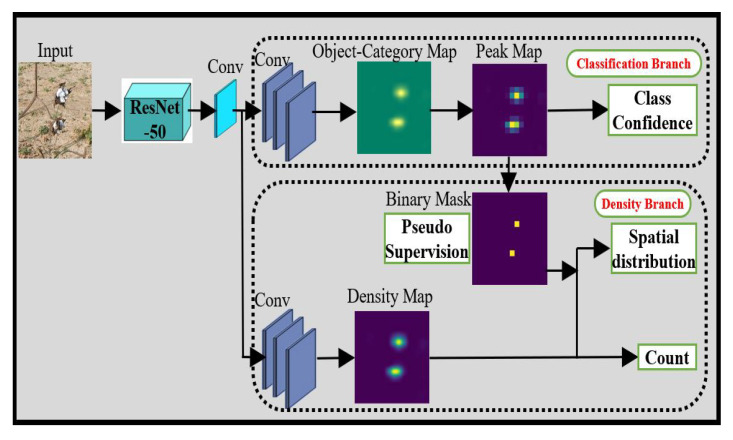
CountSeg architecture for boll counting. The two branches, classification branch and density branch, are jointly trained using image-level lower-counts (ILC) supervision. The pseudo ground truth is generated by classification branch to supervise the output of density map with the help of spatial and global loss functions.

Figure 3 illustrates the general network architecture of the CountSeg network for boll counting. The input image is passed through the ResNet-50 backbone feature extractor and 1 × 1 convolution is applied before feeding into the two main branches of this network. Out of available features, half of the features are fed to image classification branch whereas the other half are given to density branch as an input. The image classification branch performs simple convolution operation to predict the presence or absence of the boll in the image whereas the density branch is responsible for predicting the global boll count as well as its spatial distribution for that image by constructing a density map.

CountSeg architecture is jointly trained end to end with the help of image-level lower-counts (ILC) supervision [38]. The main objective of the joint training is to minimize the total loss that consists of three loss terms (Equation (2)).

(2) $$L=L_{\mathrm{class}}+L_{\mathrm{spatial}}+L_{\mathrm{global}}$$

The term Lclass denotes the loss of the classification branch which is trained with the supervised class labels. The class labels were extracted from point labels, where a count greater than zero was considered as presence label and count equal to zero was given an absence label. The peaks generated from the classifier alone contain a large number of false positives. To address the issue, the lower-level count information is incorporated to generate the pseudo ground truth for training the density branch. The term Lglobal helps to reduce the error between predicted count and ground truth count whereas the term Lspatial ensures that all the individual object instances are localized properly. Detailed implementation of these loss functions can be found at [19].

**Figure 4:**
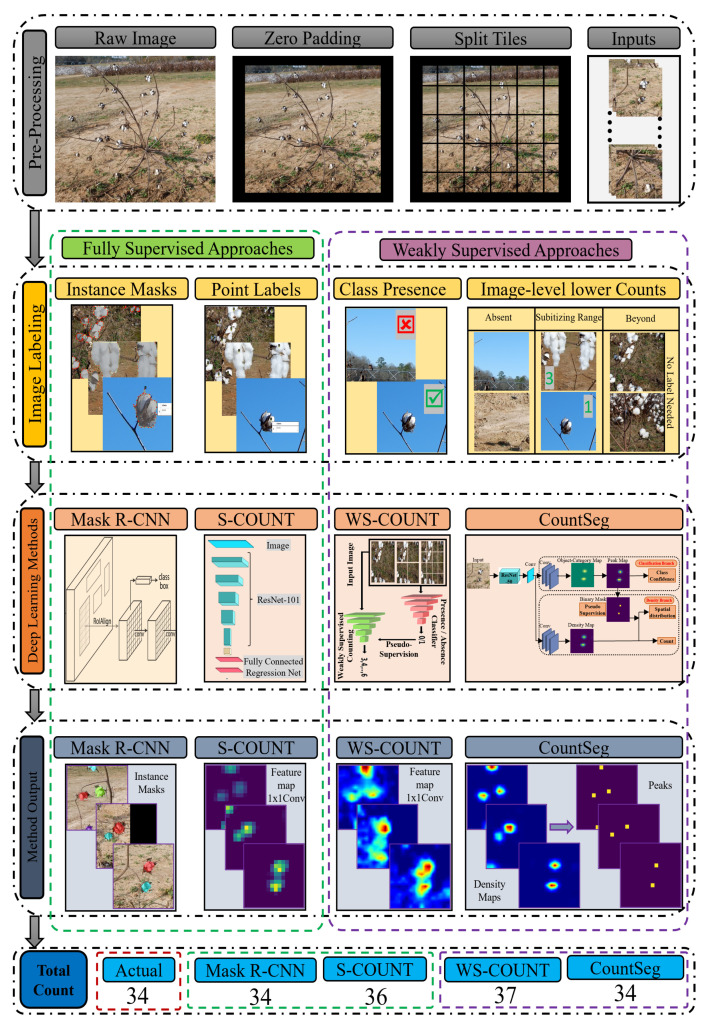
Overview of the boll counting workflow.The image tiles generated after pre-processing were labelled with point and mask labels. The classification labels (✓and ×) and image-level boll counts (1, 2, 3…) were derived from point label counts. Two fully supervised and two weakly supervised counting methods were trained on the image tiles’ training set (Table 2). The intermediate and final stage output of each methods can be visualized by instance masks and feature maps that will be used to obtain final boll count. In this example, the raw image (top row) contains 34 cotton bolls which were predicted accurately by both the Mask R-CNN and CountSeg Methods.

Figure 4 shows the overall workflow for boll counting. The full-scale raw images were pre-processed into image tiles (Section 2.1), which were annotated with instance masks, point labels, or binary (presence or absence) labels. The classification labels and image-level counts were derived from point label counts. Two fully supervised (Mask R-CNN and S-Count) and two weakly supervised (WS-Count and CountSeg) counting methods were trained on the respective annotated image tiles. The intermediate and final stage output of each method can be visualized by instance masks and feature maps that are used to obtain the final boll count. The total count for an entire plant image was calculated as the sum of counts from all the tiles. Although the question of counting a single boll multiple times may arise, very few instances of such case were observed in the raw dataset. To verify this, the 5 held out images were tested in an end-to-end fashion.

### 2.5. Boll Counting

### 2.6. Evaluation Metrics

The performance of boll counting from the two supervised (Mask R-CNN and S-Count) and two weakly supervised models (WS-Count and CountSeg) was compared in terms of counting accuracy and annotation efficiency. The boll counting task was evaluated on validation and test sets explained in Table 2 with 200 and 84 image tiles in each set, respectively. To eliminate any random errors, each of the four models was trained five times by changing random initialization and data shuffling. The evaluation methods include:

**(1) Absolute counting error and root mean squared error.** Counting error is the difference between the ground truth and predicted count, which was visualized with an error histogram. As each model was trained five times with varying prediction results, the error for a single image was computed by taking the median of the five predictions.

In addition to the absolute counting error, the root mean squared error (RMSE) (Equation (3)) was computed to measure the spread of the error. RMSE provides the same unit (i.e., boll count) as the absolute error and penalizes outliers more compared to absolute error, which is desirable in this case for counting bolls in a smaller range.

(3) $$RMSE=\sqrt{MSE}=\sqrt{\frac{1}{N}\sum_{i=1}^{N}{\left(Groundtruth\;count_{i}-Prediction\;count_{i}\right)}^{2}}$$

**(2) Linear regression.** Linear regression analyses with least squares method were conducted to evaluate the correlation between the predicted and the ground truth. The fitted line was compared with the standard Y = X line along with corresponding R^2^ values.

As several image tiles have the same boll count, the scatter plot took the form of a bubble plot, in which the radius of the bubble depicted the number of samples representing the corresponding point.

**(3) Annotation time.** One of the purposes of this study was to obtain accurate and precise boll counts with minimum annotation effort. While annotating the image tiles, the time required was manually recorded for each of the annotation approaches by a single annotator. These measurements can be used to compare the performance of supervised and weakly supervised approaches with respect to the efforts to properly train the models.

### 2.7. Implementation Details

The Mask R-CNN network was built using Matterport’s [39] tensorflow implementation and the further experiments were performed by tuning the hyperparameters with a small sample size. To keep the model simple, ResNet-50 was used as feature extractor with pre-trained weights on COCO dataset. The remaining parameters were kept unchanged from the original implementation. The model was trained using SGD optimizer and a fixed learning rate of 0.001. The 300 training images were augmented with the help of image augmentation library [40]. The augmentations were randomly selected from flip, rotate, blur, and scale operations. Another supervised model, the S-Count network, was trained with the counts obtained from point labels with the feature map size, *N* = 6 to 8. Two weakly supervised models, WS-Count and CountSeg methods, were primarily implemented based on the codes provided by the authors [41,42] with a modified feature map layer to fit the boll data. For the classifier PAC and entire WS-Count model, *N* = 6 was observed to perform better and thus used for further experiments. At first, the PAC was trained with the help of class labels extracted from the point labels to yield a robust classifier that can be used for supervising WS-Count architecture. To train the WS-Count network, SGD and Adam optimizers were tested with a learning rate of 0.0001. The rest of the parameters were unchanged from original implementation. For CountSeg, ResNet-50 was used as the backbone feature extractor to keep the model more lightweight than the original implementation. In addition to replacing the backbone, the size of channels for the 1 × 1 convolution filter was chosen to be 60, as it offered significant counting performance gain. The network was optimized using SGD optimizer with a learning rate of 0.001. All of the above mentioned models were trained on computer cluster nodes with Nvidia Tesla P100-PCIE-16GB GPU cards at Georgia Advanced Computing Resource Center (GACRC) [43].

## 3. Results and Discussion

### 3.1. Model Performance on Boll Counting Accuracy

**Figure 5:**
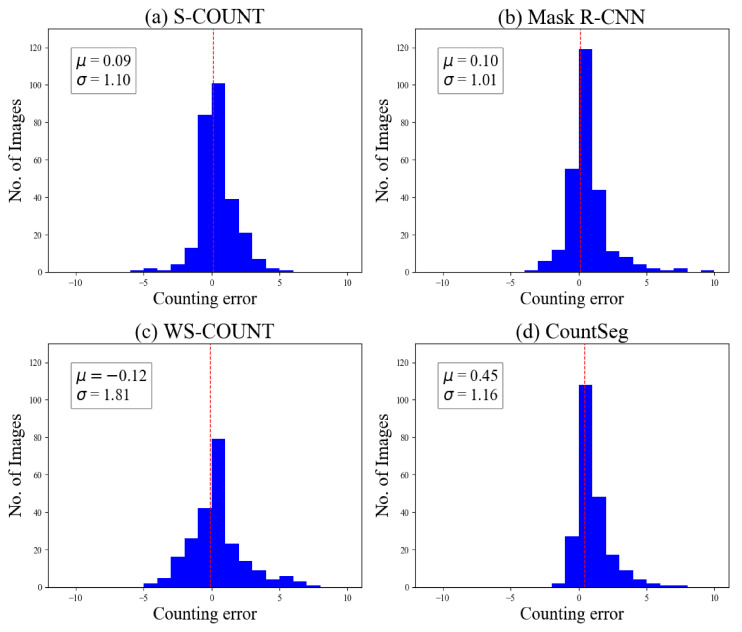
Error histograms from median predictions given by each method. Error is computed as the difference between the ground truth count and median of predicted counts from five model variations.

The histogram of error distribution revealed that the mean errors of the two supervised models (S-Count and Mask R-CNN) were closer to zero and had less spread of error than those of the two weakly supervised models (Figure 5). For example, the majority (99%) of counting errors for the supervised methods were within ±3, whereas only 93% of the images had the counting error within this range for weakly supervised methods. However, the spread of errors for CountSeg are comparable to that for both the supervised methods. Errors on the positive side signified the under-counting by the methods within a certain range. This can be attributed to the fact that the weakly supervised models were not directly trained on actual boll counts but were trained on the presence or absence of bolls.

If the data contained fixed-sized objects and the subitizing range could be increased, then this under-counting could be reduced. However, in the case of bolls, the images in this dataset had large variations in boll size, shape, and depth that limited the subitizing range to [0, 10].

**Figure 6:**
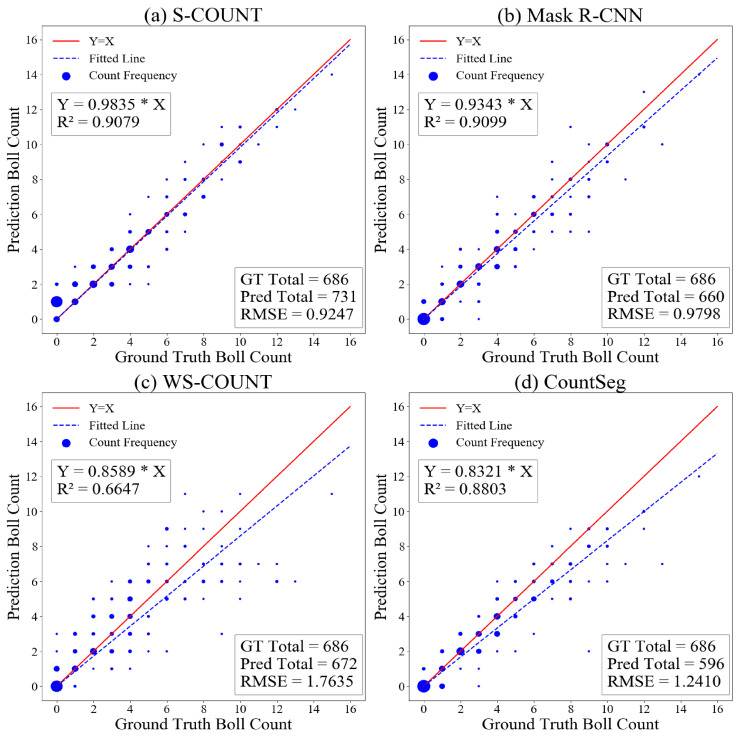
Bubble plots and linear regression between ground truth and predicted boll counts. The total boll count from 200 validation images is shown to demonstrate counting capabilities of the method.

The linear regression analyses (Figure 6) show that although the supervised methods performed better than weakly supervised methods, CountSeg achieved a comparable performance with Mask R-CNN and S-Count. The WS-Count network that combines the MB-PAC and S-Count (a regression network) learns to regress the total count with the help of PAC and thus performs according to expectations.

Comparisons of mean RMSE of the four models on the test set revealed that the weakly supervised methods performed well within the subitizing range ([0, 10]) but were far less accurate than the supervised methods when boll counts were greater than ten (Table 3). It should be noted that as the boll number grew, all the methods showed more errors as the images with higher numbers of bolls were likely to contain occlusions, closely placed bolls, or visually challenging instances that were hard to detect without any depth information. Overall, the mean RMSE values of the weakly supervised methods were close to the supervised methods despite being trained with a minimum amount of supervision.

**Figure 7:**
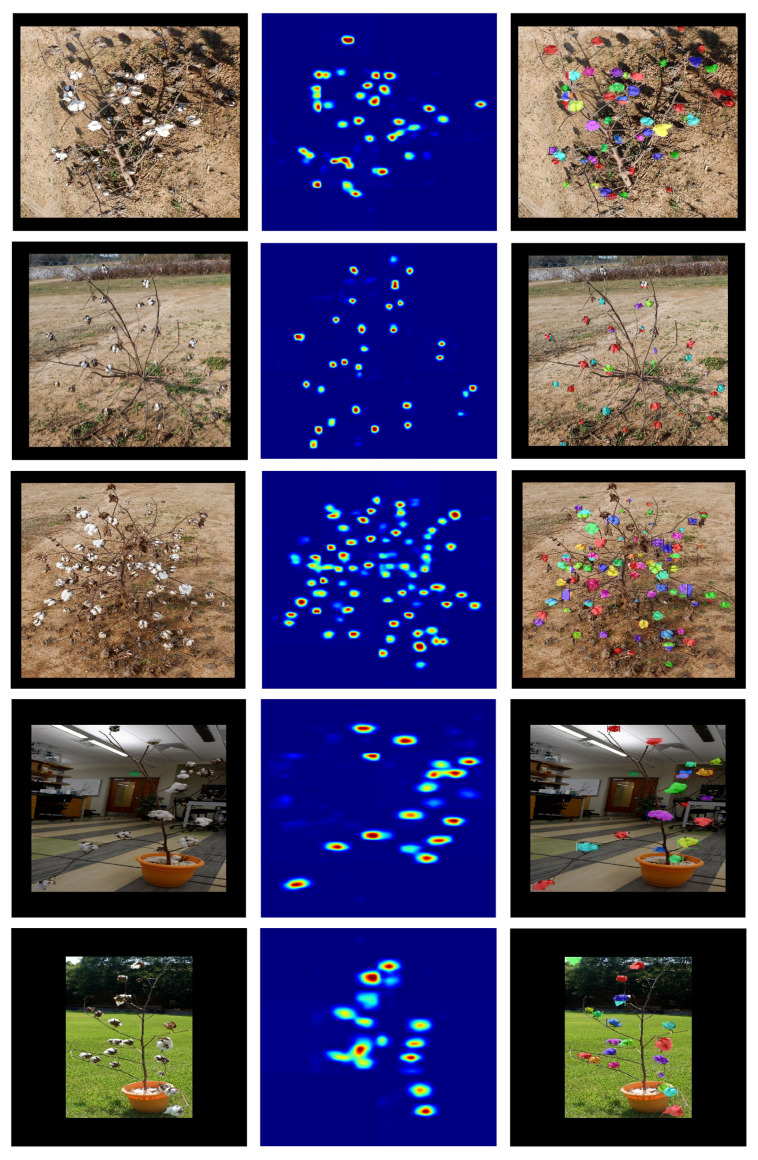
Comparison of CountSeg and Mask R-CNN. This shows the output from CountSeg density maps and prediction instance masks from Mask R-CNN for 5 held-out test samples (starting from the top row): Boll_008, Boll_022, Boll_041, Boll_116, Boll_127, respectively. It can be observed that even with lower supervision, CountSeg was able to retain the spatial contexts for most of the bolls.

To verify the performance of the end-to-end counting approach, these models were compared with respect to the total boll count on full scale images (Table 4). The outputs of one weakly supervised method (CountSeg) and one supervised method (Mask R-CNN) were illustrated on images representing the dataset of in-field and potted plants taken under different conditions (Figure 7). For example, image Boll_008 was taken from a tilted angle such that the plant casts shadows of the bolls on the ground, creating a false boll instance in the image. In this case, all of the methods show random performance as the boll count varies largely for each model. Supervised methods, such as Mask R-CNN, overestimated the count because of the false predictions of masks to shadows, whereas CountSeg underestimated the number because of occluded bolls forming a single peak. Image Boll_022 depicts an irregularly shaped cotton plant that has visually distinguishable cotton bolls and every patch in the image has a different background texture. The top part of the image has bolls on the far background, which produce noisy input, and all the models misdetected a few of those bolls in the background as true bolls belonging to the plant in final predictions. Nevertheless, because of the clear separation between bolls, one weakly supervised method (CountSeg) and Mask R-CNN give predictions close to the actual ground truth. In fact, CountSeg yielded better counts on all 5 of its model variations than S-Count and Mask R-CNN.

One of the challenges for this end-to-end processing pipeline was to handle crowded bolls in a single image, as can be seen in image Boll_041. All the methods underestimated the total count for the image because this image is visually difficult for counting in 2D viewpoint. Nevertheless, the counts obtained from WS-Count show least variation and are close to the predictions of supervised counts. Extending the application to indoor potted plants (Boll_116), both supervised and weakly supervised methods show highly precise counting performance. The accuracy is slightly lower as the background and illumination contribute to the noise in the image, which lead to a reduced spatial context that can be used to separate boll instances. On the other hand, potted images in outdoor conditions (Boll_127) are slightly easier to process as the boll instances are less occluded by the background and illumination. In conclusion, supervised methods performed slightly better in adverse image conditions but the weakly supervised methods without adequate spatial context during training were able to localize most of the boll instances, even in adverse conditions such as noisy background, large boll population, and changing illuminations. The CountSeg method produces highly accurate density maps which can be combined with a proposal ranking method to achieve weakly supervised instance segmentation (Figure 7). The instance masks produced by CountSeg can be compared with the masks obtained from Mask R-CNN, thereby replacing the intensive supervised method with a low-cost weakly supervised method.

### 3.2. Annotation Time Comparison

**Figure 8:**
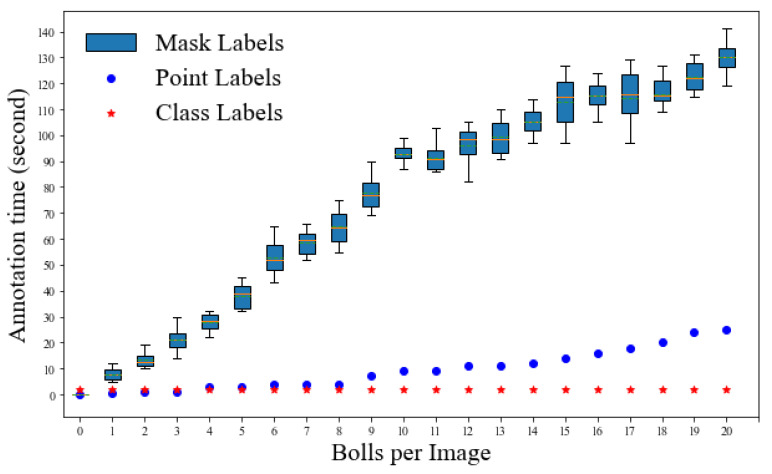
Comparison of annotation time of three types of labels. The time taken for annotating an image tile was measured with respect to the boll count in that image tile. A sample set of 10 images per boll count was considered and the average times were reported for point labels whereas the box plots represent the range of time taken by mask labels for the same boll count.

Average annotation time was compared among three annotation methods (mask, point, and class labels) and the class annotation method showed a clear advantage (Figure 8). For annotating instance masks, the annotator must draw exact polygons that cover all the pixels in that instance. As a result, the time required to annotate a single instance varies according to the size, shape, and visual separability from other boll instances. The time required to annotate an image increases significantly as the number of bolls in the image increases. In the case of point labels, the annotator is required to click/draw a single point for a single instance, which can be done in a comparable amount of time for a fixed number of bolls per image. The time required to annotate an image increases moderately as the number of bolls in the image increases. The class labels, in contrast, are the simplest form of annotation in which the annotator can simply select “yes” or “no” for the presence or absence of the object instance, respectively. Irrespective of the boll count per image, on average class labels require approximately 2 s per image.

It can be seen that point labels are at least 10 times faster for the images with boll counts in the subitizing range ([0, 10]) and at least 15 times faster for images beyond the counting range than the mask labels. This essentially allows researchers to use more raw data in the training process given the fixed amount of time. In terms of boll counting, the performance gap between fully supervised and weakly supervised seems acceptable, considering the huge advantage with respect to annotation costs and the potential to significantly increase the training data. Experimentation with current weakly supervised methods to improve the spatial consistency, such as the use of the Generative Adversarial Networks (GAN) along with the WS-Count architecture, may result in an even better performance in the future [44].

### 3.3. Discussion

This study investigated both the supervised deep learning methods and weakly supervised learning approaches for cotton boll counting from low-cost RGB images with satisfactory results. Previous studies used traditional image processing techniques or machine learning methods that required hand-crafted features [15,18]. Many customized parameters and computationally intensive methods, such as shape transforms, split-and-merge, and density-based spatial clustering (DB-SCAN) in the previous studies make these algorithms less robust for testing on different datasets. In contrast, our approaches can learn the features automatically in an end-to-end fashion and are robust on unseen datasets.

Our study revealed that supervised and weakly supervised approaches had different annotation costs. With minimum efforts spent in data annotations, weakly supervised methods achieved comparable performance with a supervised approach under similar inference conditions. Furthermore, the intermediate results obtained from the peak response maps could easily be used for the semantic segmentation problem, and, if provided with object proposals, the peak response maps can perform instance segmentation at the annotation cost of classification.

On the other hand, while working with weakly supervised methods for boll detection, a few questions remain open for discussion, such as occlusion handling, identifying instances in high density of cotton bolls, and real-time in-field detection of bolls. Supervised segmentation of cotton bolls showed better performance in dealing with occlusions and counting densely populated cotton bolls as they learn from precise instance boundaries. However, the proposed weakly supervised methods do not have the instance boundaries from which to learn, so instead they aggregate the peaks observed in the boll feature maps, making them prone to under-counting in such scenarios. The advancements in current WS-Count architecture [44] to handle high density and occlusions can help answer those questions with a few modifications in the current experimental setup. Furthermore, because the data annotated for weakly supervised methods is easy to reproduce and can be reused across a variety of different algorithms, one can try an ensemble of these methods to obtain the best match for their counting task, for example, boosting the boll counting weak detector with a pre-trained source, that was trained for a similar task, with the help of knowledge transfer [45]. This may help to solve the problem of the dense population of cotton bolls and the occlusions caused by other organs through the incorporation of the instance boundary knowledge of a strong base learner.

Three-dimensional point cloud data encompass the missing depth information from 2D images and thus are considered as an alternative to address the 2D overlapping structures and occlusions by other plant parts [46]. For instance, 3D point clouds obtained from a cotton plant can be segmented with the help of deep learning to accurately segment the organs to perform more intricate trait extraction [47]. However, 3D data collection typically involves more expensive instruments (such as LiDAR) and takes a longer time to process (such as the photogrammetry methods) than RGB images. Furthermore, pre-processing and post-processing of 3D data require more skills and computational resources. Considering these factors, the proposed method using RGB images is a low-cost and effective way to perform the initial field analysis and to estimate boll counts.

## 4. Conclusions

In this work, both the supervised and weakly supervised deep learning models achieved promising results in cotton boll counting from proximal images. With the help of manually annotated datasets for segmenting and counting cotton bolls, a supervised boll instance segmentation pipeline was developed and it achieved excellent segmentation performance. Weakly supervised approaches were tested and achieved slightly lower performance in boll counting than the supervised models, but their annotation cost was 10 times lower than that of supervised methods. This significant saving in annotation cost makes it possible to train a large dataset to boost the performance of the weakly supervised learning models. Future work will be directed toward training models with high resolution data and measuring more fine-grained cotton plant and boll phenotypic traits such as main stalk, node number, branch angle, and internode distance. The supervised and weakly supervised deep learning models for boll counting with low-cost RGB images can be used by cotton breeders, physiologists, and growers alike to improve crop breeding and yield estimation.

## Figures and Tables

**Table 1:** Summary of raw images selected for boll counting task and corresponding tiles generated after zero-padding along with sum of boll count per tile. A total of 285 full plant images were selected to obtain image tiles for training and testing of weakly supervised methods with random shuffling. The remaining 5 full plant images were held out to test all the methods on a full scale plant.

Image Set	No. of Images	No. of Tiles Generated	No. of Bolls
Training + Testing	285	4266	23,651
Full Plant Testing	5	84	217
**Total**	**290**	**4350**	**23,868**

**Table 2:** Summary of boll count per tile in different counting ranges for each of the train, validation, and test datasets. Train and validation set contain tiles obtained from 285 full-scale raw images whereas test set represents tiles from 5 held-out images. Because of the dense boll population, the tiles containing more than 15 bolls cannot be counted accurately. Hence, these were not considered (DNC) in training or in the total count for the training and validation sets.

Boll Count per Image	Training Tiles	Validation Tiles	Testing Tiles	Total
**0**	919	50	21	990
**[1, 5]**	1852	102	48	2002
**[6, 10]**	710	42	6	758
**[11, 15]**	231	6	3	240
**above 15**	300 (DNC)	54 (DNC)	6	360
**Total**	3712	200	84	4350

**Table 3:** Testing set boll counting mean RMSE along with standard deviation for different methods with respect to boll count per image.

Boll Count/Image	0	[1–5]	[6–10]	[11–15]	Total
**Train/Test split**	919/50	1852/102	710/42	231/6	**3712/200**
**S-Count**	0.582 ± 0.25	1.069 ± 0.16	1.556 ± 0.26	2.430 ± 1.04	**1.181 ± 0.16**
**WS-Count**	0.708 ± 0.07	1.431 ± 0.25	2.489 ± 0.23	5.314 ± 0.39	**1.826 ± 0.05**
**CountSeg**	0.286 ± 0.06	0.869 ± 0.02	1.978 ± 0.14	3.805 ± 0.45	**1.284 ± 0.08**
**Mask R-CNN**	0.566 ± 0.20	0.982 ± 0.04	1.586 ± 0.42	2.884 ± 1.03	**1.175 ± 0.20**

**Table 4:** Hold-out test set average boll count with std. deviation for the five models of each method. These images represent the entire plant under various conditions.

Image ID	Boll_008	Boll_022	Boll_041	Boll_116	Boll_127
**Actual Count**	41	34	100	20	22
**S-Count**	43.6 ± 4.22	41.8 ± 11.12	87.8 ± 7.50	16.4 ± 1.67	21.4 ± 1.67
**WS-Count**	48.2 ± 1.31	40.2 ± 3.49	86.4 ± 2.79	23.2 ± 1.48	18.8 ± 1.30
**CountSeg**	37.8 ± 2.49	**34.2 ± 0.84**	81.8 ± 3.56	17.0 ± 0.00	18.0 ± 1.414
**Mask R-CNN**	46.0 ± 6.16	34.8 ± 1.90	**89.0 ± 3.94**	**17.2 ± 0.84**	**21.2 ± 0.45**

## Data Availability

The dataset used in this study can be accessed at the following link: https://doi.org/10.6084/m9.figshare.19665096.v1.
